# Lightweight and Efficient Authentication and Key Distribution Scheme for Cloud-Assisted IoT for Telemedicine

**DOI:** 10.3390/s25092894

**Published:** 2025-05-03

**Authors:** Hyang Jin Lee, Sangjin Kook, Keunok Kim, Jihyeon Ryu, Hakjun Lee, Youngsook Lee, Dongho Won

**Affiliations:** 1 Department of Electrical and Computer Engineering, Sungkyunkwan University, Suwon-si 16419, Republic of Korea; hyangjin.lee@gmail.com (H.J.L.); sangjinkook@gmail.com (S.K.); kimkeunok@gmail.com (K.K.); dhwon@security.re.kr (D.W.); 2 School of Computer and Information Engineering, Kwangwoon University, Seoul-si 01897, Republic of Korea; 3 School of Electronic Engineering, Kumoh National Institute of Technology, Gumi-si 39177, Republic of Korea; hjlee@kumoh.ac.kr; 4 Department of Computer, Howon University, 64 Impi-myeon, Howondae 3-gil, Gunsan-si 54058, Republic of Korea; ysooklee@howon.ac.kr

**Keywords:** cloud-assisted IoT, lightweight and efficient authentication, IoT for telemedicine

## Abstract

Medical Internet of Things (IoT) systems are crucial in monitoring the health status of patients. Recently, telemedicine services that manage patients remotely by receiving real-time health information from IoT devices attached to or carried by them have experienced significant growth. A primary concern in medical IoT services is ensuring the security of transmitted information and protecting patient privacy. To address these challenges, various authentication schemes have been proposed. We analyze the authentication scheme by Wang et al. and identified several limitations. Specifically, an attacker can exploit information stored in an IoT device to generate an illegitimate session key. Additionally, despite using a cloud center, the scheme lacks efficiency. To overcome these limitations, we propose an authentication and key distribution scheme that incorporates a physically unclonable function (PUF) and public-key computation. To enhance efficiency, computationally intensive public-key operations are performed exclusively in the cloud center. Furthermore, our scheme addresses privacy concerns by employing a temporary ID for IoT devices used to identify patients. We validate the security of our approach using the formal security analysis tool ProVerif.

## 1. Introduction

Medical Internet of Things (IoT) services facilitate communication between doctors and patients for monitoring and treating health conditions [1,2]. The COVID-19 pandemic has further accelerated the global demand for telemedicine, expanding medical IoT services beyond hospitals [3,4,5]. While the demand for such services varies by country, they have become particularly important in nations with large elderly populations [6,7], enabling patients to remotely monitor their health and connect with distant hospitals. According to a 2025 report, the global telemedicine market is projected to reach USD 107.52 billion in 2024, USD 121.10 billion in 2025, and USD 432.1 billion by 2032 [8].

Typically, telemedicine services begin with the pre-registration of the patient, attending physician, IoT medical device, and gateway in the hospital’s cloud center [9]. The patient’s health data are then regularly transmitted to the hospital’s cloud center via a gateway (GW) installed at home or in a nearby common location [10]. The physician reviews these data and provides appropriate responses.

In telemedicine services, IoT devices attached to or carried by patients have significantly fewer resources than medical devices within hospitals [11]. Additionally, to collect, process, and manage large volumes of patient data from diverse locations, a high-performance cloud center is essential. Thus, telemedicine imposes additional requirements beyond those of hospital-based medical IoT environments. However, owing to the sensitivity of transmitted data, ensuring mutual authentication among participants and protecting privacy and anonymity are critical. In recent years, various studies have explored these challenges in medical IoT environments.

More recently, authentication schemes have also been proposed that use cloud servers with unlimited computing resources to process information sent from a large number of IoT devices. These authentication schemes can be used in telemedicine, where a large amount of patient information needs to be processed. In 2023, C. Wang et al. [12] proposed a user authentication scheme for cloud-assisted IoT. However, this paper describes the limitation that this authentication scheme can lead to illegitimate session key exchange if an attacker obtains information stored in the user’s smart device or IoT device, which can lead to sensor node impersonation attack. In this paper, we propose a lightweight and efficient authentication scheme that overcomes the limitations of C. Wang et al. [12] and apply it to telemedicine services that used medical IoT devices with limited computing resources, and enhance privacy and anonymity. The key contributions of the proposed scheme are summarized as follows.

- We analyze C. Wang et al.’s work in [12] and find that their proposed authentication scheme is vulnerable to a stolen mobile device attack, which can lead an attacker to generate an illegitimate session key. We also show that the proposed scheme fails to achieve the goal of using cloud centers to increase efficiency by allowing IoT devices to perform public-key cryptographic computation.
- We propose an authentication scheme using cloud centers with unlimited computing resources, similar to the one proposed by C. Wang et al. in [12]. However, unlike C. Wang et al. [12], our scheme improves the efficiency of the authentication scheme by performing public-key cryptographic computation (ECC) only for users (smart devices) and cloud centers that have a certain level of computing resources.
- The proposed scheme minimizes the computation at IoT devices by using a physically unclonable function (PUF). In the scheme, the PUF’s challenge–response pair is transmitted through a private channel during the IoT device registration phase, and only the PUF’s challenge is transmitted through a public channel during the authentication and key distribution phases, making it resistant to PUF modeling attacks.
- The proposed scheme is resistant to user impersonation attacks, stolen-device attacks, PUF modeling attacks, etc., and provides anonymity and untraceability, mutual authentication, and forward and backward secrecy.
- The proposed scheme is verified using ProVerif, an official security analysis tool. In terms of performance, the proposed scheme achieves 35,436.18% computational savings compared to other recent studies, particularly on low-capacity sensor nodes.

The remainder of this paper is organized as follows: Section 2 discusses related works. Section 3 presents the system model for telemedicine and the attack model. Section 4 and Section 5 introduce Wang’s scheme and its identified vulnerabilities, respectively. Section 6 details the proposed authentication and key distribution scheme. Section 7 and Section 8 provide formal and informal security analyses, along with security and efficiency evaluations. Finally, Section 9 concludes the paper.

## 2. Related Work

With the advancement of IoT technology, numerous recent studies have proposed user authentication methods to ensure secure connections between IoT devices and users.

In 2018, Wazid et al. introduced the user-authenticated key management protocol (UAKMP), a secure and lightweight three-factor remote user authentication scheme for hierarchical IoT networks comprising various nodes, such as gateway nodes, cluster head nodes, and sensing nodes [13]. UAKMP provides password update functionality, ensures anonymity, and supports offline sensing node registration. However, according to Wang et al. [12], it does not satisfy clock synchronization and fails to guarantee forward secrecy.

Several authentication schemes have also been proposed for industrial IoT (IIoT) environments [14,15]. In 2020, Srinivas et al. introduced a user authentication scheme enabling remote users to analyze data in IIoT environments [15]. Their scheme employs biometric information, smart cards, and passwords for authentication, supporting password updates and smart card revocation in cases of loss or theft. However, according to Wang et al. [12], it does not ensure user anonymity. Similarly, in 2020, Yang et al. proposed a dynamic authentication credential framework for IIoT environments [14]. Their scheme achieved faster computation by avoiding public-key cryptography; however, according to Wang et al. [12], it does not guarantee forward secrecy.

Furthermore, in 2020, Wazid et al. proposed a user authentication scheme for smart-home environments [16]. Their scheme achieved high computational efficiency by utilizing only symmetric encryption and decryption. However, according to Wang et al. [12], it fails to ensure forward secrecy.

Recently, authentication schemes targeting a broader range of service environments have been proposed. In 2022, Dai et al. [17] proposed an authentication scheme for multigateway sensor networks. The scheme in [17] reduces communication requirements between gateways by registering two frequently visited gateway nodes and improves authentication efficiency using ECC. In the same year, Hu et al. [18] proposed a cloud-assisted authentication scheme based on Chebyshev polynomial encryption for IIoT environments. The scheme in [18] leverages cloud-computing technology with unlimited computing resources to reduce IoT device computation time. Additionally, in [19,20], cloud-computing technology has been utilized for authentication and key sharing in vehicle networks.

In 2023, Wang et al. [12] proposed a user authentication scheme for IoT using cloud centers. However, ref. [12] is vulnerable to stolen mobile devices and user-impersonation attacks, which may result in illegal session key exchanges. Our proposed scheme addresses the limitations of [12] and proposes a lightweight and efficient authentication and key distribution protocol.

## 3. Preliminaries

In this section, we introduce the system and attack models of the proposed scheme, along with the definitions and properties of the techniques used.

### 3.1. System Model

**Figure 1:**
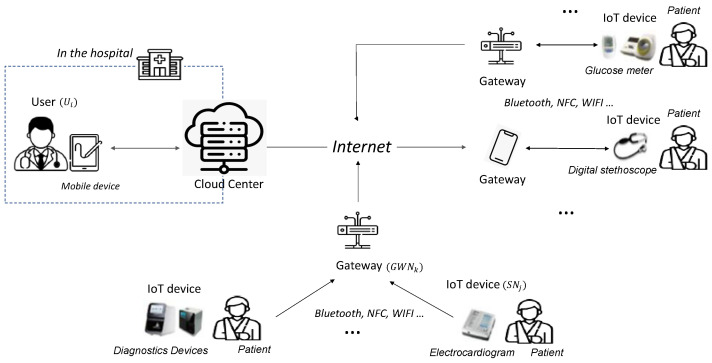
System model for telemedicine.

The proposed scheme targets a telemedicine system model, as shown in Figure 1, and consists of four entities: the user, cloud center, gateway, and IoT device. In this model, a doctor and their mobile device within a hospital are defined as the user, while a medical device that collects patient health information is classified as an IoT device. The gateway transmits data from the IoT device to the cloud center, which serves as a trusted hub for collecting, monitoring, managing, and processing patient information before transmitting it to the user. Each participant is described in detail below.

1. User ($U_{i}$): The user $U_{i}$ is a legitimate entity with access to the IoT device. In this scheme, $U_{i}$ is a doctor who remotely monitors a patient’s health through an IoT device attached to the patient. As the system is designed for telemedicine, $U_{i}$ accesses the IoT device through the CloudCenter. Communication with the CloudCenter occurs via a smart device such as a smartphone or tablet.
2. IoT Device ($SN_{j}$): The IoT device $SN_{j}$ collects medical and health information from the patient and transmits it to $U_{i}$ through the gateway $GWN_{k}$ and the CloudCenter. $SN_{j}$ is typically a low-power medical device placed in or on the patient’s body, such as a glucose meter or digital stethoscope. Therefore, it is assumed that $SN_{j}$ cannot perform complex, resource-intensive operations. To enhance security, the proposed method uses physically unclonable function (PUF) technology in $SN_{j}$.
3. Gateway ($GWN_{k}$): The gateway $GWN_{k}$ is a trusted entity responsible for transmitting information collected from $SN_{j}$ to the CloudCenter. While $GWN_{k}$ has greater computing power than $SN_{j}$, it faces limitations in handling large volumes of data transmitted by numerous IoT devices in a telemedicine environment. $GWN_{k}$ mainly communicates with $SN_{j}$ using Bluetooth, NFC, or Wi-Fi and connects to the CloudCenter via the Internet.
4. Cloud Center (CloudCenter): The CloudCenter is a trusted entity responsible for processing, managing, storing, and distributing patient information collected from multiple gateways to users. It plays a critical role in telemedicine services, requiring substantial computing resources to handle vast amounts of data from remotely located IoT devices. In the proposed scheme, the cloud center facilitates authentication and key distribution by securely sharing secret parameters during the registration process of $U_{i}$, $GWN_{k}$, and $SN_{j}$. It is assumed that the CloudCenter is securely managed and protected from attackers. In the cloud-assisted IoT for telemedicine services proposed in this paper, the cloud center does not play the role of a simple gateway that supports mutual authentication between users and IoT devices but is proposed as a centralized scheme to perform monitoring and analysis of patient information collected from IoT devices using the computing resources of the cloud center. In particular, in telemedicine services, patients (IoT devices) are often assigned to a doctor in advance, and the doctor has to manage a large number of patients, so a centralized scheme is a more realistic service model than a decentralized or peer-to-peer scheme.

### 3.2. Attacker Model

We assume that the cryptographic primitives used in the proposed scheme are secure and the attacker has the following capabilities:

1. According to the Dolev–Yao model [21,22,23,24,25], the attacker can eavesdrop and intercept the messages transmitted over the public communication channel. Also, the attacker can delete, modify, and reply to any message transmitted over the public channel.
2. The attacker can physically capture the user’s smart device, gateway, or IoT device and obtain all stored information, including certain security parameters.
3. The attacker cannot access information stored in the Cloud Center.
4. The Cloud Center and Gateway each securely hold their long-term secrets, and an attacker cannot obtain them.

### 3.3. Elliptic Curve Cryptography

ECC is a public-key cryptosystem that utilizes the mathematical properties of elliptic curves to achieve secure encryption [26,27,28]. It provides strong security with a smaller key size compared to traditional public-key cryptosystems such as RSA, offering improved performance. The proposed scheme is based on the elliptic curve discrete logarithm problem (ECDLP), which states that given a point *P* and a base point *G* (∈Zp) on an elliptic curve Zp, it is computationally difficult to determine an integer d such that P=d·G.

### 3.4. Fuzzy Extractor

A fuzzy extractor is a cryptographic technique designed to generate a stable and secure cryptographic key from biometric data or other noisy inputs [28,29]. As biometric measurements [30], such as fingerprints, may exhibit slight variations between scans, a fuzzy extractor ensures that the same cryptographic key is consistently derived despite these minor differences. It consists of two main procedures: a probabilistic generation procedure (Gen) and a deterministic reproduction procedure (Rep), characterized as follows:

- Gen takes a biometric input BIO and generates a random string δ along with an auxiliary string τ, Gen(BIO)=(δ,τ).
- Rep takes as input BIO, a similar $BIO^{*}$, and the auxiliary string τ generated by Gen, and reconstructs the random string δ, $Rep(BIO^{*},\tau )=\delta$.

### 3.5. Physically Unclonable Function, PUF

A PUF is a hardware-based security primitive that leverages inherent physical variations in semiconductor manufacturing to generate a unique, unclonable cryptographic key [31,32]. These unpredictable manufacturing variations produce distinct responses for each chip, making replication or cloning infeasible. A PUF operates based on the challenge–response pair (CRP) model, where an input challenge generates a unique response based on the physical characteristics of the hardware. When the same challenge is applied to the same chip, it consistently produces the same response, whereas different chips generate entirely different responses.

In this study, we adopt PUFs to achieve both lightweight operation and strong security for sensor nodes. PUFs generate random values based on intrinsic physical characteristics without requiring additional storage or heavy computation, unlike hardware security modules such as TPMs or symmetric key cryptographic approaches. Moreover, compared to HMAC-based schemes or traditional symmetric-key methods, PUFs offer stronger resistance against hardware cloning attacks. Due to these properties, PUFs are particularly suitable for resource-constrained medical IoT environments where computational capability and power consumption are critical concerns. PUFs are assumed to satisfy the following conditions:

1. PUFs are physically unclonable: PUFs are inherently resistant to cloning and modification. They are also highly resistant to environmental factors and aging. An identical device cannot be reproduced by cloning a PUF, and any attempt to change a device containing a PUF will modify its functionality or render it unusable.
2. PUFs exhibit one-way function properties: Given a response Rn from a specific PUF, it is computationally infeasible to derive the corresponding challenge Cn.
3. Different PUFs generate different responses: For the same challenge Cn, two different PUFs (PUF1 and PUF2) will produce distinct responses Rn1 and Rn2.
4. A PUF produces a consistent response for the same challenge: A given PUF always outputs the same response Rn when presented with the same challenge Cn.
5. Low probability of response collision among multiple PUFs: The likelihood of two different PUFs generating identical responses is extremely low. This property enables PUFs to serve as unique identifiers for distinguishing a large number of IoT devices.

The proposed scheme applies PUFs to the IoT device $SN_{j}$. Using PUF properties, $SN_{j}$ uses PUF during the registration phase with the CloudCenter and $GWN_{k}$, as well as during the authentication and session key distribution phase with $GWN_{k}$.

## 4. Review of Wang et al.’s Scheme

In this section, the scheme introduced by Wang et al. [12] is described. The scheme consists of four participants: the user, cloud center, gateway, and IoT device (sensor node). The user and IoT device perform mutual authentication to share a session key using the cloud center. The scheme is divided into four phases: gateway and IoT device registration, user registration, login, and authentication. The details are as follows:

### 4.1. Gateway and IoT Device Registration Phase

This phase comprises the registration of the gateway and IoT device with the cloud center. The details are as follows:

#### 4.1.1. Gateway Registration

To register with the cloud center CloCen, the gateway $GWN_{k}$ follows these steps:

1. $GWN_{k}$ sends a registration request to CloCen with $GID_{k}$.
2. CloCen computes $X_{G_{k}}=h\left(x\|GID_{k}\right)$ using its secret key *x* and sends the message $GID_{k},\;X_{G}k$ to $GWN_{k}$.
3. $GWN_{k}$ stores $X_{G_{k}}$.

#### 4.1.2. IoT Device Registration

To register with $GWN_{k}$, the IoT device $S_{j}$ follows these steps:

1. $GWN_{k}$ sends a registration request to CloCen with $SID_{j}$.
2. $GWN_{k}$ validates $SID_{j}$. If it is valid, $GWN_{k}$ computes $X_{S_{j}}=h\left(SID_{j}\|x_{G_{k}}\right)$, using $GWN_{k}$ ’s secret key $x_{G_{k}}$. $GWN_{k}$ then sends $SID_{j},X_{S_{j}}$ to $S_{j}$.
3. $S_{j}$ keeps $X_{S_{j}}$ as its private key.

### 4.2. User Registration Phase

1. $U_{i}$ inputs their identity $ID_{i}$, password $PW_{i}$, and biometric information $Bio_{i}$ into their smart mobile device. The device selects a random number $a^{\prime }$, and computes $Gen\left(Bio_{i}\right)=\left(\delta_{i},\;\tau_{i}\right)$, $RPW_{i}=h(PW_{i}\|\delta_{i}\left\|a^{\prime }\right)$. The registration request $\left\{ID_{i},\;RPW_{i}\right\}$ is then sent to CloCen.
2. CloCen selects a timestamp $T_{rg_{i}}$ and a random number $a_{i}$, then computes $k_{i}=h(ID_{i}\left\|y\right\|T_{rg_{i}})$, $B_{i}^{\prime }$ = $h\left(RPW_{i}\|ID_{i}\right)⊕k_{i}$. The CloCen stores $\left\{ID_{i},T_{rg_{i}},a_{i},Honeylist=NULL\right\}$ in its database and sends $\left\{B_{i}^{\prime },B_{i}^{\prime }⊕a_{i},\;Y,\;P\right\}$ to $U_{i}$.
3. Upon receiving $\left\{B_{i}^{\prime },B_{i}^{\prime }⊕a_{i},Y,P\right\}$, the smart device selects a new random number *a* and calculates the following values. Finally, the smart device stores $k_{i}$, $RPW_{i}^{new}$, $A_{i}$, $B_{i}$, $a_{i}$ as $\left\{A_{i},B_{i},a,A_{i}⊕a_{i},\tau_{i},Y,P,n0\right\}$.
  -
$k_{i}=B_{i}^{\prime }⊕h\left(RPW_{i}\|ID_{i}\right)$

  -
$RPW_{i}^{new}=h(PW_{i}\|\delta_{i}\left\|a\right)$

  -
$A_{i}=h(ID_{i}\|RPW_{i}^{new}\left\|k_{i}\right)modn0$

  -
$B_{i}=h\left(RPW_{i}^{new}\|ID_{i}\right)⊕k_{i}$

  -
$a_{i}=B_{i}^{\prime }⊕\left(B_{i}^{\prime }⊕a_{i}\right)$

### 4.3. Login Phase

If $U_{i}$ wants to access an IoT device, it initiates a login request to $GWN_{k}$ as follows:

1. $U_{i}$ enters $ID_{i}^{*},PW_{i}^{*},Bio_{i}^{*}$ into the smart device, which then computes $\delta_{i}^{*}=Rep\left(Bio_{i}^{*},\tau_{i}\right)$, $RPW_{i}^{*}=h(PW_{i}^{*}\|\delta_{i}^{*}\left\|a\right)$, $k_{i}^{*}=B_{i}⊕RPW_{i}^{*}$, $A_{i}^{*}=h(ID_{i}^{*}\|RPW_{i}^{*}\left\|k_{i}^{*}\right)modn0$. The device then compares $A_{i}^{*}$ with $A_{i}$ to verify the authenticity of $U_{i}$. If $A_{i}^{*}$ is not equal to $A_{i}$, the login request is rejected.
2. If $A_{i}^{*}$ is equal to $A_{i}$, the smart device selects a random number $r_{i}$ and computes $a_{i}^{*}$, M1, M2, M3, M4, and M5. The device then sends M2, M3, M4, M5 to CloCen.
  - $a_{i}^{*}=\left(A_{i}⊕a_{i}\right)⊕A_{i}$
  - $M1=r_{i}\cdot Y$, $M2=r_{i}\cdot P$
  - $M3=h\left(M2\|M1\right)⊕\left(ID_{i}^{*}\|a_{i}^{*}\right)$
  - $M4=h\left(M1\|M2\|M3\right)⊕SID_{j}$
  - $M5=h(k_{i}^{*}\|ID_{i}^{*}\|M1\|M2\|SID_{j})$

### 4.4. Authentication Phase

1. To verify the $U_{i}$, CloCen computes $M1^{\prime }=y\cdot M2$, $ID_{i}^{\prime }\|a_{i}^{\prime }=M3⊕h\left(M2\|M1^{\prime }\right)$. It then retrieves $\left\{Trg_{i},\;a_{i}\right\}$ using $ID_{i}^{\prime }$. If $a_{i}$ is equal to $a_{i}^{’}$, CloCen computes $k_{i}^{\prime }=h(ID_{i}^{\prime }\left\|y\right\|T_{rg_{i}})$, $SID_{j}^{\prime }=M4⊕h\left(M1\|M2\|M3\right)$, $M5^{\prime }=h(k_{i}^{\prime }\|ID_{i}^{\prime }\|M1^{\prime }\|M2\|SID_{j}^{\prime })$. It then verifies $U_{i}$ via $M5^{\prime }$. If $M5^{\prime }$ is equal to M5, CloCen accepts the authenticity of $U_{i}$.
2. CloCen inserts $k_{i}$ into Honeylist if there are fewer than 10 items. If the Honey−list exceeds 10, $U_{i}$’s account is suspended until re-registration. CloCen then determines the gateway $GWN_{k}$ to which $S_{j}$ belongs, selects a random number *r*, and computes $X_{G_{k}}^{\prime }$, M6, M7, and M8 as shown below and sends M2, M6, M7, M8 to the gateway node $GWN_{k}$.
  -
$X_{G_{k}}^{\prime }=h\left(x\|GID_{k}\right)$

  -
$M6=h(X_{G_{k}}^{\prime }\|M2)⊕r$

  -
$M7=h(M6\|r\|X_{G_{k}}^{\prime })⊕SID_{j}^{\prime }$

  -
$M8=h(M2\|M6\|M7\|r\|SID_{j}^{\prime }\|X_{G_{k}}^{\prime })$

3. $GWN_{k}$ computes $r^{\prime }=M6⊕h\left(X_{G_{k}}\|M2\right)$, $SID_{j}^{\prime \prime }=M7⊕h(M6\|r^{\prime }\left\|X_{G_{k}}\right)$, $M8^{\prime }=h(M2\|M6\|M7\|r^{\prime }\|SID_{j}^{\prime \prime }\left\|X_{G_{k}}\right)$, and then verifies whether $M8^{\prime }?=M8$. If $M8^{\prime }$ is equal to M8, $GWN_{k}$ computes $X_{S_{j}}^{\prime }$, M9, M10 as shown below, and sends M2,M9,M10.
  -
$X_{S_{j}}^{\prime }=h\left(x_{G_{k}}\|SID_{j}^{\prime \prime }\right)$

  - $M9=h(X_{S_{j}}^{\prime }\|M2⊕r_{g}$ (where $r_{g}$ is a random number chosen by $GWN_{k}$)
  -
$M10=h(M2\|M9\|r_{g}\|SID_{j}^{\prime \prime }\|X_{S_{j}}^{\prime })$

4. The IoT device $S_{j}$ computes $r_{g}^{\prime }=M9⊕h\left(X_{S_{j}}\|M2\right)$, $M10^{\prime }=h(M2\|M9\|r_{g}^{\prime }\|SID_{j}\left\|X_{S_{j}}\right)$, and compares the values of $M10^{\prime }$ and M10. If $M10^{\prime }$ is equal to M10, $S_{j}$ chooses a random number $r_{j}$, calculates $M=r_{j}\cdot M2$, $M11=r_{j}\cdot P$, SK=h(M2‖M11‖M), $M12=h(M2\|M11\|r_{g}^{\prime }\|X_{S_{j}}\|SID_{j})$, and responds {M11,M12} to the gateway $GWN_{k}$.
5. $GWN_{k}$ computes $M12^{\prime }=h(M2\|M11\|r_{g}\|X_{S_{j}}^{\prime }\left\|SID_{j}^{\prime \prime }\right)$ and compares $M12^{\prime }$ with M12 to verify the identity of $S_{j}$. If $M12^{\prime }$ is equal to M12, $GWN_{k}$ calculates $M13=h(M11\|M2\|SID_{j}^{\prime \prime }\|r^{\prime }\|X_{G_{k}})$ and sends {M11,M13} to CloCen.
6. CloCen computes $M13^{\prime }=h(M11\|M2\|SID_{j}^{\prime \prime }\left\|r\right\|X_{G_{k}}^{\prime })$ to test the identity of $GWN_{k}$. If $M13^{\prime }$ is equal to M13, CloCen computes $M14=h(M1^{\prime }\|M2\|ID_{i}^{\prime }\|SID_{j}^{\prime \prime }\|k_{i}^{\prime }\|M11)$, and then returns {M11,M14} to $U_{i}$.
7. Having obtained CloCen’s reply {M11,M14}, the smart mobile device computes $M14^{*}=h(M1\|M2\|ID_{i}\|SID_{j}\left\|k\|M11\right)$. If $M14^{*}$ is equal to M14, $U_{i}$ accepts $SK=h(M2\|M11\|r_{i}\cdot M11)$ as his session key shared with $S_{j}$, and the authentication process finishes successfully.

## 5. Limitations of Wang et al.’s Scheme

We identified several critical limitations in Wang et al. [12]’s scheme. A detailed analysis of these vulnerabilities is provided below.

### 5.1. Stolen Mobile Device Attack

In Wang et al. [12]’s scheme, only legitimate users are allowed to log in using $PW_{i}$ and $Bio_{i}$ during the user login phase. However, after user $U_{i}$ logs in, the messages M1, M2, M3, M4, and M5, which authenticate the user to CloCen, contain only information stored in the smart device. Although Wang et al. [12]’s scheme includes $ID_{i}$ as a credential known only to the user, in general, user IDs in various Internet protocols are publicly accessible for identification, or they can often be computed in polynomial time by an attacker. This implies that if an attacker gains access to the user’s smart device and retrieves its stored information, they can successfully authenticate with CloCen without requiring the login phase. The following is a detailed attack scenario.

- {In step 2 of Section 4.3 Login Phase, the attacker computes $a_{i}$ using the information $A_{i}$ and $A_{i}⊕a_{i}$ stored in the stolen mobile device, and generates a random number $r_{a}$. In addition, the attacker can find the $ID_{i}$ of the legitimate user in polynomial time.
- The attacker then generates the following message using the previously calculated $a_{i}$, $ID_{i}$, and $r_{a}$ to impersonate the legitimate user, and sends it to CloCen:
  – $M1_{a}=r_{a}\cdot Y$ (where Y is ECC public key of CloCen)
  – $M2_{a}=r_{a}\cdot P$ (where P is ECC base point of CloCen)
  –
$M3_{a}=h\left(M2_{a}\|M1_{a}\right)⊕\left(ID_{i}\|a_{i}\right)$

  –
$M4_{a}=h(M1_{a}\|M2_{a}\left\|M3_{a}\right)⊕SID_{j}$

  –
$M5_{a}=h(k_{i}\|ID_{i}\|M1_{a}\|M2_{a}\left\|SID_{j}\right)$

- In step 2 of Section 4.4 Authentication Phase, CloCen verifies $M1_{a}=y\cdot M2_{a}$ using its private key y. If $M1_{a}$ is equal to $y\cdot M2_{a}$, then it finds $ID_{i}$ through $ID_{i}\|a_{i}=M3_{a}⊕h\left(M2_{a}\|M1_{a}\right)$.
- The attacker can be authenticated as a legitimate user because all subsequent steps use the information stored inside CloCen regarding $ID_{i}$ to perform subsequent authentication procedures.

### 5.2. Illegitimate Session Key Exchange

If the attacker knows $SID_{j}$ and obtains $X_{S_{j}}$ from a captured IoT device, they can manipulate the session key exchange, as shown below.

- In step 4 of Section 4.4 Authentication Phase, the attacker chooses a random number $r_{j_{a}}$ and calculates $M_{a}$, $M11_{a}$ using the transmitted message M2 as follows.
  –
$M_{a}=r_{j_{a}}\cdot M2$

  – $M11_{a}=r_{j_{a}}\cdot P$ (where P is the ECC base point of CloCen)
- The attacker uses the previously computed $M_{a}$, $M11_{a}$, and the transmitted message M2 to generate an illegitimate session key $SK_{a}$. The attacker also generates additional information $M12_{a}$ so that the gateway and user can generate the same session key, and sends $M11_{a},\;M12_{a}$ to $GWN_{k}$.
  –
$SK_{a}=h(M2\|M11_{a}\left\|M_{a}\right)$

  – $M12_{a}=h(M2\|M11_{a}\|r_{g}^{’}\|X_{S_{j}}\left\|SID_{j}\right)$ (where $r_{g}^{’}$ is a random number generated by the gateway in step 3 of Section 4.4 Authentication Phase, and $X_{S_{j}}$ is $h(SID_{j}\|x_{G_{k}})$. And $x_{G_{k}}$ is the long-term secret of $GWN_{k}$.)
- In step 5 of Section 4.4 Authentication Phase, $GWN_{k}$ checks if $M12_{a}$ is equal to $h(M2\|M11_{a}\|$$r_{g}$‖$X_{S_{j}}$$\|SID_{j})$ using $M11_{a}$ and $M12_{a}$ sent by the attacker and the information M2, $r_{g}$, $X_{S_{j}}$, and $SID_{j}$ that it knows, where $X_{S_{j}}$ is $h(SID_{j}\|x_{G_{k}})$. If it is equal, $GWN_{k}$ computes $M13_{a}=h\left(M11_{a}\right\|$$M2\|SID_{j}\left\|r\right\|X_{G_{k}})$ and sends $\left\{M11_{a},\;M13_{a}\right\}$ to CloCen.
- In step 6 of Section 4.4 Authentication Phase, CloCen checks whether $M13_{a}$ is equal to $h(M11_{a}\|M2\|SID_{j}\|r\|X_{G_{k}})$ using $M11_{a}$ and $M13_{a}$ sent from $GWN_{k}$ and the information M2, $SID_{j}$, *r*, and $X_{G_{k}}$ that it knows, where $X_{G_{k}}$ is $h(x\|GID_{k})$, and *x* is the secret long-term key of CloCen. If it is equal, CloCen computes $M14_{a}=h(M1\|M2\|ID_{i}$$\|SID_{j}\|k_{i}\left\|M11_{a}\right)$ and sends $\left\{M11_{a},\;M14_{a}\right\}$
- In step 7 of Section 4.4 Authentication Phase, $U_{i}$ uses $M11_{a}$ and $M14_{a}$ sent by CloCen and the information M1, M2, $ID_{i}$, $SID_{j}$, $k_{i}$, and $M11_{a}$, knowing to check whether $M14_{a}$ matches $h(M1\|M2\|ID_{i}\|SID_{j}\|k_{i}\|M11_{a})$. If it matches, the user generates a session key $SK_{a}$ as follows.
  – $SK_{a}=h(M2\|M11_{a}\left\|r_{i}\cdot M11_{a}\right)$ (where, $r_{i}\cdot M11_{a}=r_{j_{a}}\cdot M2=M_{a}$)

Through this process, the illegally generated session key $SK_{a}$ is exchanged between $U_{i}$ and $SID_{j}$ by the attacker.

### 5.3. Inefficiency

A key contribution of Wang et al.’s scheme is the use of cloud-computing technology to overcome the computational and storage limitations of gateways and IoT devices. Reducing the computational load on the gateway or IoT devices significantly impacts the overall efficiency of the authentication scheme. However, in Wang et al.’s scheme, both the gateway and IoT devices perform computationally intensive ECC operations. Therefore, it is unclear whether the scheme effectively reduces the computational load on these resource-constrained devices compared to other authentication schemes that rely on symmetric key algorithms, despite the incorporation of cloud-computing technology.

## 6. Proposed Scheme

In this section, we propose a secure and efficient authentication and key distribution scheme to establish a secure telemedicine environment by addressing the vulnerabilities in Wang et al.’s scheme [12] described in Section 5 and integrating a cloud-assisted IoT framework into the medical domain.

**Figure 2:**
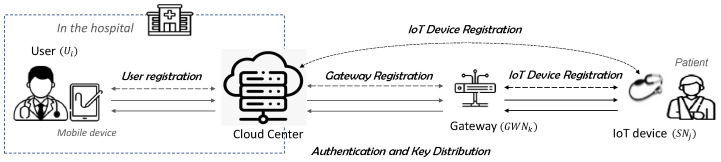
Proposed authentication scheme.

The proposed scheme is applied to the system model shown in Figure 2. In this model, a doctor in a hospital serves as the user ($U_{i}$), while a medical IoT device (sensor node, $SN_{j}$) attached to or carried by the patient monitors the patient’s condition outside the hospital. The patient’s health data are transmitted from the IoT device to the cloud center through a gateway ($GWN_{k}$), enabling the user to access the transmitted information, assess the patient’s condition, and provide appropriate prescriptions remotely.

To use the telemedicine service, the patient must first register the user $U_{i}$, who is the doctor in charge, along with the medical IoT device $SN_{j}$, and the gateway $GWN_{k}$ with the CloudCenter. Therefore, in our proposed scheme, it is assumed that the cloud center is aware of the user $UID_{i}$ and gateway $GID_{k}$ based on the IoT device $SID_{j}$ before the registration phase. In addition, in the proposed scheme, the user $U_{i}$ establishes a session key with the CloudCenter, while $GID_{k}$ and $SID_{j}$ share their respective session keys with the CloudCenter through mutual authentication. Consequently, direct authentication between $UID_{i}$, $GID_{k}$, and $SID_{j}$ is not required. The notation used in the proposed scheme is provided in Table 1.

### 6.1. User Registration Phase

**Figure 3:**
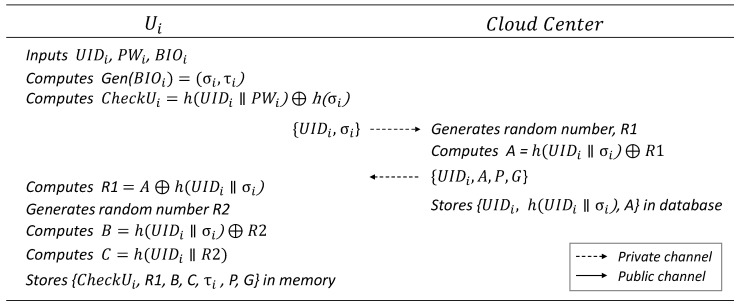
User registration phase.

In this phase, $U_{i}$ enters a user ID $UID_{i}$, password $PW_{i}$, and biometric information $BIO_{i}$ on the mobile device to initiate a legitimate login and register validation information with the CloudCenter for authentication. The details are illustrated in Figure 3.

1. $U_{i}$ inputs $UID_{i}$, $PW_{i}$, and $BIO_{i}$ into the mobile device, computes $Gen\left(BIO_{i}\right)=\left(\delta_{i},\tau_{i}\right)$, and generates $CheckU_{i}=h(UID_{i}\|PW_{i}\left\|\right)⊕h\left(\delta_{i}\right)$ as additional validation information to prove legitimacy. $U_{i}$ then sends $\left\{UID_{i},\;\delta_{i}\right\}$ to the CloudCenter through a secure private channel.
2. The CloudCenter generates a random number R1 and computes $A=h(UID_{i}\|\delta_{i})$. It then sends $\left\{UID_{i},A,P,G\right\}$ to $U_{i}$, where *G* is the base point of the shared ECC algorithm, and *P* is the public key of the CloudCenter. After registering $U_{i}$, the CloudCenter stores $\left\{UID_{i},h\left(UID_{i}/parallel\delta_{i}\right),A\right\}$ in its database.
3. $U_{i}$ computes $R1=A⊕h(UID_{i}\|\delta_{i})$ and generates a random number R2. It then computes $B=h(UID_{i}\|\delta_{i})⊕R2$, $C=hH(UID_{i}\|R2)$ and stores $\left\{CheckU_{i},R1,B,C,\tau_{i},P,G\right\}$ in the mobile device’s memory.

### 6.2. Gateway Registration Phase

**Figure 4:**
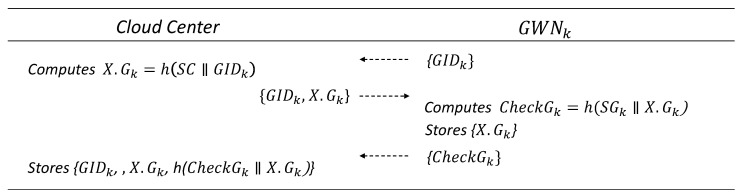
Gateway registration phase.

In this phase, $GWN_{k}$ and the CloudCenter complete the registration process using their respective long-term secrets, $S_{C}$ and $S_{G_{k}}$, which are securely held by $GWN_{k}$ and CloudCenter, respectively. It is assumed that the CloudCenter is already aware of $GID_{k}$ and $SID_{j}$, through prior offline registration. The details are illustrated in Figure 4.

1. $GWN_{k}$ sends a registration request message containing $GID_{k}$ to CloudCenter.
2. CloudCenter computes $X_{G_{k}}=h\left(S_{C}\|GID_{k}\right)$ and sends $\left\{GID_{k},X_{G_{k}}\right\}$ to $GWN_{k}$.
3. $GWN_{k}$ computes $CheckG_{k}=h\left(SG_{k}\|X_{G_{k}}\right)$ and sends it to the cloud center. It then stores $/X_{G_{k}}/$ in memory.
4. $GWN_{k}$ stores $\left\{GID_{k},X_{G_{k}},h\left(CheckG_{k}\|X_{G_{k}}\right)\right\}$ in its database.

### 6.3. IoT Device Registration Phase

**Figure 5:**
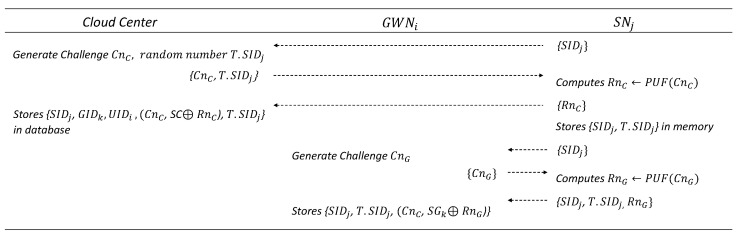
IoT device (sensor) registration phase.

In this phase, $SN_{j}$ registers with CloudCenter and $GWN_{k}$, respectively. The details are illustrated in Figure 5.

#### 6.3.1. IoT Device Registration to Cloud Center

1. $SN_{j}$ sends a registration request message containing $SID_{j}$ to CloudCenter.
2. CloudCenter identifies $SN_{j}$ using $SID_{j}$ and generates a challenge $Cn_{C}$ along with a random number $T.SID_{j}$, where $T.SID_{j}$ is a randomly generated temporary ID for $SID_{j}$. The CloudCenter then sends $\left\{Cn_{C},\;T.SID_{j}\right\}$ to $SN_{j}$.
3. $SN_{j}$ generates a response $Rn_{C}$ for $Cn_{C}$ and sends it to the CloudCenter. After sending the response, $SN_{j}$ stores $\left\{SID_{j},\;T.SID_{j}\right\}$.

#### 6.3.2. IoT Device Registration to Gateway

1. $SN_{j}$ sends a registration request message containing $SID_{j}$ to $GWN_{k}$.
2. $GWN_{k}$ generates a challenge $Cn_{G}$ and sends it to $SN_{j}$.
3. $SN_{j}$ computes the response $Rn_{G}\leftarrow PUF\left(Cn_{G}\right)$ and sends $\left\{SID_{j},\;T.SID_{j},\;Rn_{G}\right\}$ to $GWN_{k}$.
4. $GNW_{k}$ stores $\left\{SID_{j},\;T.SID_{j},\;\left(Cn_{G},\;SG_{k}⊕Rn_{G}\right)\right\}$ in its database.

### 6.4. Authentication and Key Distribution Phase

**Figure 6:**
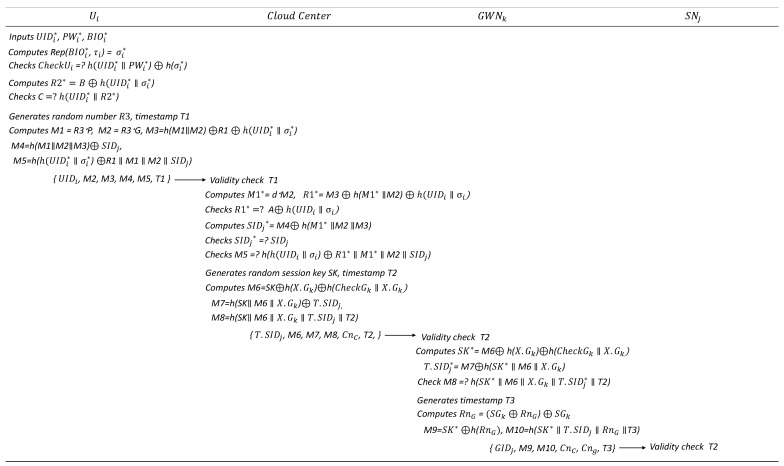
Authentication and key distribution phase (Steps 1–3).

**Figure 7:**
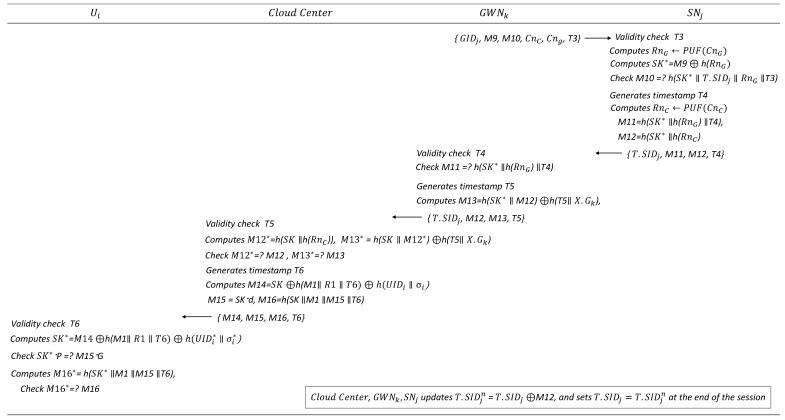
Authentication and key distribution phase (Steps 4–7).

In the proposed scheme, authentication and key distribution among the four participants are managed centrally by the CloudCenter. User $U_{i}$ does not communicate directly with $GWN_{k}$ or $SN_{j}$; instead, all authentication and key exchange processes are facilitated through the cloud center. The details are illustrated in Figure 6 and Figure 7.

1. User $U_{i}$ begins the authentication process by entering $UID_{i}^{*}$, $PW_{i}^{*}$, and $BIO_{i}^{*}$ on the mobile device, which then computes $\delta_{i}^{*}=Rep\left(BIO_{i}^{*},\tau_{i}\right)$. The device verifies whether $h\left(UID_{i}^{*}\|PW_{i}^{*}\right)⊕h\left(\delta_{i}^{*}\right)$ is equal to $CheckU_{i}$ stored on it. If the check passes, the device computes $R2^{*}=B⊕h\left(UID_{i}^{*}\|\delta_{i}^{*}\right)$ and further verifies whether $h(UID_{i}^{*}\|R2*)$ is equal to *C*. If successful, $U_{i}$ is logged into the mobile device. The device then generates a random number R3 and timestamp T1, computing M1, M2, M3, M4, and M5 as follows:
  - M1=R3·P, M2=R3·G, $M3=h\left(M1\|M2\right)⊕R1⊕h\left(UID_{i}^{*}\|\delta_{i}^{*}\right)$
  -
$M4=h\left(M1\|M2\|M3\right)⊕SID_{j}$

  -
$M5=h(h\left(UID_{i}\|\delta_{i}\right)⊕R1\|IM1\|M2\|SID_{j}))$

  Finally, $U_{i}$ sends $\left\{UID_{i},\;M2,\;M3,\;M4,\;M5,\;T1\right\}$ to CloudCenter.
2. Upon receiving the message, the CloudCenter first verifies the validity of the timestamp T1. If it is invalid, the protocol terminates; otherwise, the CloudCenter retrieves $UID_{i}$ and its associated information from its database. It then computes $M1^{*}=d\cdot M2$, $R1^{*}=M3⊕h\left(M1^{*}\|M2\right)⊕h\left(UID_{i}\|\delta_{i}\right)$, checking whether $A⊕h(UID_{i}\|\delta_{i})$ matches $R1^{*}$. If this condition is met, the CloudCenter computes $SID_{j}^{*}=M4⊕h\left(M1*\|M2\|M3\right)$ and verifies whether it matches $SID_{j}$. To authenticate $U_{i}$, it further checks whether $h(h\left(UID_{i}\|\delta_{i}\right)⊕R1^{*}\|M1^{*}\|M2\|SID_{j})$ matches M5. If successful, the user authentication is complete. Then CloudCenter sends the messages $\left\{T.SID_{j},M6,M7,M8,T2,Cn_{C}\right\}$ to $GWN_{k}$. The CloudCenter then identifies the target gateway $GID_{k}$, generates a random session key SK, and a timestamp T2, and computes the following: M6, M7, and M8.
  -
$M6=SK⊕h\left(X_{G_{k}}\right)⊕h\left(CheckG_{k}\|X_{G_{k}}\right)$

  -
$M7=h(SK\|M6\|X_{G_{k}})⊕T.SID_{j}$

  -
$M8=h(SK\|M6\|X_{G_{k}}\|T.SID_{j}\|T2)$

  Finally, the CloudCenter sends $\left\{T.SID_{j},\;M6,\;M7,\;M8,\;T2,\;Cn_{C}\right\}$ to $GWN_{k}$.
3. Upon receiving the message from the CloudCenter, $GWN_{k}$ first verifies the validity of timestamp T2. If invalid, the protocol terminates; otherwise, $GWN_{k}$ computes $SK^{*}=M6⊕h\left(X_{G_{k}}\right)⊕h\left(CheckG_{k}\|X_{G_{k}}\right)$, $T.SID_{j}^{*}=M7⊕h(SK^{*}\left\|M6\right\|X_{G_{k}})$ and verifies whether $h(SK^{*}\|M6\|X_{G_{k}}\|T.SID_{j}^{*}\|T2)$ matches M8. If successful, $GWN_{k}$ generates timestamp T3, and computes $Rn_{g}$, M9, and M10 as follows:
  -
$Rn_{g}=\left(S_{G_{k}}⊕Rn_{g}\right)⊕S_{G_{k}}$

  -
$M9=SK⊕h(Rn_{G})$

  -
$M10=h(SK\|T.SID_{j}\|Rn_{g}\|T3)$

  $GWN_{k}$ then sends $\left\{GID_{k},\;M9,\;M10,\;T3,\;Cn_{C},\;Cn_{G}\right\}$ to $SN_{j}$.
4. Upon receiving the message, $SN_{j}$ verifies the validity of timestamp T3. If invalid, the protocol terminates; otherwise, $SN_{j}$ computes $Rn_{G}\leftarrow PUF\left(Cn_{G}\right)$, $SK^{*}=M9⊕h\left(Rn_{G}\right)$ and verifies whether $h(SK^{*}\|T.SID_{j}\|Rn_{G}\|T3)$ matches M10. If successful, $SN_{j}$ generates timestamp T4 and computes $Rn_{C}$, M11, and M12 as follows:
  -
$Rn_{C}\leftarrow PUF\left(Cn_{C}\right)$

  -
$M11=h(SK^{*}\|h\left(Rn_{g}\right)\|T4)$

  -
$M12=h(SK^{*}\|h\left(Rn_{C}\right))$

  $SN_{j}$ then sends the message $\left\{T.SID_{j},\;M11,\;M12,\;T4\right\}$ to $GWN_{k}$.
5. $GWN_{k}$ verifies the validity of timestamp T4 and verifies whether $h(SK\|h\left(Rn_{G}\right)\|T4)$ matches M11. If valid, $GWN_{k}$ generates timestamp T5 and computes $M13=h\left(SK\|M12\right)⊕h\left(T5\|X_{G_{k}}\right)$, then sends $\left\{T.SID_{j},\;M12,\;M13,\;T5\right\}$ to the CloudCenter.
6. Upon receiving the message, the CloudCenter verifies the validity of timestamp T5. If valid, it retrieves $SID_{j}$, $GID_{k}$, and $UID_{i}$ from the database regarding $T.SID_{j}$ and computes $M12^{*}=h\left(SK\|h\left(Rn_{C}\right)\right)$, $M13^{*}=h\left(SK\|M12^{*}\right)⊕h\left(T5\|X_{G_{k}}\right)$. The CloudCenter then verifies whether M12 and M13 match $M12^{*}$ and $M13^{*}$, respectively. If successful, it generates timestamp T6 and computes M14, M15, and M16 as follows:
  -
$M14=SK⊕h\left(M1\|R1\|T6\right)⊕h\left(UID_{i}\|\delta_{i}\right)$

  -
M15=SK·d

  -
M16=h(SK‖M1‖M15‖T6)

  The CloudCenter sends the message {M14,M15,M16,T6} to $U_{i}$.
7. $U_{i}$ verifies the validity of timestamp T6. If valid, $U_{i}$ computes $SK^{*}$ and confirms the integrity of the shared $SK^{*}$ by performing the following steps:
  - Computing $SK^{*}=M14⊕h\left(M1\|R1\|T6\right)⊕h\left(UID_{i}^{*}\|\delta_{i}^{*}\right)$.
  - Verifying $SK^{*}\cdot P$ matches M15·G.
  - Calculating $h(SK^{*}\|M1\|M15\|T6)$ and checking whether it is equal to M16.
  If all conditions hold, the session key is securely shared among the participants.
8. Finally, at the end of the session, the CloudCenter, $GWN_{k}$, and $SN_{j}$ update $T.SID_{j}$ as follows:
  -
$T.SID_{j}^{new}=T.SID_{j}⊕M12$

  -
$T.SID_{j}=T.SID_{j}^{new}$

### 6.5. Password Update Phase

At the end of the session, $U_{i}$ inputs $UID_{i}^{*}$, $PW_{i}^{*}$, and $BIO_{i}^{*}$ into the smart device to initiate the password update process. The device computes $CheckU_{i}^{*}=h\left(UID_{i}^{*}\|PW_{i}^{*}\right)/plush\left(\delta_{i}^{*}\right)$ and verifies whether $CheckU_{i}^{*}$ matches the stored $CheckU_{i}$. If the values match, $U_{i}$ inputs $PW_{i}^{new}$ as the new password and computes $CheckU_{i}^{new}=h\left(UID_{i}\|PW_{i}^{n}\right)/oplush\left(\delta_{i}\right)$. Finally, the password update is completed when $U_{i}$ updates $CheckU_{i}$ to $CheckU_{i}^{new}$ and stores it in the device’s memory.

## 7. Security Analysis of Proposed Scheme

In this section, we present both formal and informal security analyses. The details are as follows:

### 7.1. Formal Security Analysis

In this paper, the ProVerif tool is used to formally analyze the security of the proposed authentication protocol [33,34,35,36,37]. ProVerif, developed by Blanchet et al. [38], is widely used for verifying security properties such as confidentiality and authentication in cryptographic protocols. The proposed protocol is modeled in Applied Pi-Calculus, and its resistance to attacks is evaluated under the Dolev–Yao model [21].

The details of each code are presented in Table 2, Table 3, Table 4, Table 5, Table 6, Table 7 and Table 8. Table 2 describes the variables, events, channels, and function declarations in the ProVerif code. Table 3 presents the user registration and authentication phases. Table 4 illustrates the operation process of the cloud center, while Table 5 outlines the gateway’s operation process. Table 6 describes the operation process of the sensor node. Table 7 lists the queries used to verify the overall operation, and Table 8 presents the results obtained when the queries from Table 7 were executed.

This study conducted an automated formal verification using the ProVerif tool to evaluate the security and safety of the designed protocol.

The ProVerif queries were configured as follows:

- query inj-event(endUser(idi)) ==> inj-event(startUser(idi)) verifies that the user’s authentication completion event occurs only if the authentication initiation event has occurred (mutual authentication for users).
- query inj-event(endCloudCenter()) ==> inj-event(startCloudCenter()) verifies the correct execution of the cloud center’s authentication process.
- query inj-event(endGateway(gwi)) ==> inj-event(startGateway(gwi)) checks the correctness of the gateway’s authentication.
- query inj-event(endSensorNode(snj)) ==> inj-event(startSensorNode(snj)) checks the correctness of the sensor node’s authentication.
- query attacker(SharedKey) verifies the confidentiality of the session key, ensuring it is not accessible to the attacker.

The verification results show that all authentication and confidentiality queries returned true. This indicates that the proposed protocol successfully guarantees mutual authentication among the user, cloud center, gateway, and sensor node, and that the session key remains confidential against potential attackers. The analysis results obtained from ProVerif show that all specified security queries were proven to be true, confirming that the protocol successfully meets its intended security requirements. Specifically, the verification demonstrated that a user’s termination event always occurs after the corresponding initiation event, clearly ensuring user authentication. Additionally, the termination event at the cloud center was verified to occur strictly following its initiation event, thereby validating the reliability of the system’s communication flow. Furthermore, the gateway and sensor nodes were also confirmed to maintain correct sequential consistency between their respective initiation and termination events, securing the integrity of message exchanges. Moreover, the analysis mathematically verified that an attacker could not access or compromise the primary shared keys, highlighting the strong confidentiality provided by the protocol. Based on these comprehensive results, it can be concluded that the proposed protocol effectively fulfills critical security requirements such as authentication, confidentiality, and integrity.

### 7.2. Informal Security Analysis

The proposed scheme satisfies nine critical security requirements, including protection against user-impersonation attacks, stolen-device attacks, session key disclosure attacks, anonymity and untraceability, mutual authentication, replay attacks, forward secrecy attacks, man-in-the-middle attacks, and PUF modeling attacks [39,40,41,42]. A comparison of the latest studies and their corresponding security properties is summarized in Table 9. The detailed security properties of the proposed scheme are as follows:

#### 7.2.1. A1: Resistance to User-Impersonation Attack

The proposed scheme effectively resists user-impersonation attacks because even if an attacker intercepts the authentication messages sent to the cloud center, they cannot generate $h(UID_{i}\|\delta_{i})$, which is a critical parameter for authentication. As the cloud center must calculate $R1^{*}=M3⊕h\left(M1^{*}\|M2\right))⊕h\left(UID_{i}\|\delta_{i}\right)$ to verify the user, the inability of the attacker to reconstruct $h(UID_{i}\|\delta_{i})$ prevents further progress in the authentication process. The scheme is also resistant to stolen-device attacks.

#### 7.2.2. A2: Resistance to Stolen-Device Attack

Even if an attacker physically steals a user’s smart device and obtains stored information $\left\{checkU_{i},\;R1,\;B,\;C,\tau_{i},\;P,\;G\right\}$, they cannot generate the valid biometric information $\delta_{i}$ required for login or authentication. Furthermore, for IoT devices, even if an attacker gains access to $\left\{SID_{j},\;T.SID_{j}\right\}$ and intercepts messages transmitted over public channels, they cannot generate the response $Rn_{G}$ used for authentication, owing to the nature of the PUF. The scheme is also resistant to stolen-device attacks.

#### 7.2.3. A3: Resistance to Session Key Disclosure Attack

Messages M6 and M9, which contain session key SK information, are transmitted over a public channel. However, an attacker cannot compute SK from these messages because they cannot access $H(X_{G_{k}})$ and $Rn_{G}$, both of which are essential for deriving SK. The scheme ensures security against session key exposure attacks.

#### 7.2.4. A4: Resistance to Replay Attack

During the authentication and key distribution phase, each transmitted message includes a timestamp, and the recipient first verifies its validity. If the timestamp is invalid, the session is immediately terminated. Therefore, the proposed scheme is resistant to replay attacks.

#### 7.2.5. A5: Resistance to Man-in-the-Middle Attack

In the proposed scheme, secret information that can only be generated by each participant is used for mutual authentication between $U_{i}$, CloudCenter, $GWN_{k}$, and $SN_{j}$ during the authentication and key distribution phases. For instance, even if an attacker intercepts all messages transmitted over the public channel, they may attempt to change M2 and M3 in a message from $U_{i}$ to CloudCenter to impersonate the legitimate $U_{i}$. However, the attacker cannot generate $h(UID_{i}\|\delta_{i})$, which is uniquely generated by $U_{i}$. Therefore, the request message sent by the attacker cannot successfully authenticate as the legitimate user. In this manner, the proposed scheme is resistant to man-in-the-middle attacks, as the authentication value is generated using secret information known only to the legitimate participants, making it impossible for an attacker to alter or forge this value.

#### 7.2.6. A6: Resistance to PUF Modeling Attack

Finally, the scheme is resistant to PUF modeling attacks, which involve collecting a large number of PUF challenge–response pairs and using machine learning techniques to predict legitimate responses. In the proposed scheme, two PUF challenge–response pairs, $\left(Cn_{C},\;Rn_{C}\right)$ and $\left(Cn_{G},\;Rn_{G}\right)$, are transmitted over a private channel during the IoT device registration phase. However, during the authentication and key-sharing phases, only the PUF challenges $Cn_{C}$ and $Cn_{R}$ are transmitted over a public channel, while the responses remain private. This prevents attackers from collecting sufficient challenge–response pairs to train a predictive model, making PUF modeling attacks infeasible.

#### 7.2.7. S1: Provide Anonymity and Untraceability

Regarding anonymity and untraceability, the proposed telemedicine system model does not require user anonymity because the doctor communicates with the cloud center rather than directly with the IoT device. However, anonymity and untraceability are necessary for the patient’s IoT device. The scheme achieves this by using $T.SID_{j}$, a temporary ID for $SID_{j}$, which is randomly generated for each session and updated at the end of the session to provide anonymity and untraceability.

#### 7.2.8. S2: Provide Mutual Authentication

During the authentication and key distribution phases, $U_{i}$, CloudCenter, $GWN_{k}$, and $SN_{j}$ undergo mutual authentication. The CloudCenter authenticates $U_{i}$ by verifying that $R1^{*}$ and M5 match $A⊕h(UID_{i}\|\delta_{i})$ and $h(h\left(UID_{i}\|\delta_{i}\right)⊕R1^{*}\|M1^{*}\|M2\|SID_{j})$, respectively. $GWN_{k}$ authenticates the CloudCenter by verifying M8, while $SN_{j}$ authenticates $GWN_{k}$ by verifying M10. If any of these values are invalid, the session is aborted, ensuring that the proposed scheme provides mutual authentication.

#### 7.2.9. S3: Provide Forward and Backward Secrecy

The proposed scheme guarantees forward and backward secrecy by ensuring that the session key SK is randomly generated for each session. Although it is used to compute M12, it cannot be inferred owing to the one-way nature of the hash function. Therefore, even if the session key for a particular session is compromised, an attacker cannot determine the session keys for previous or future sessions. Therefore, the proposed scheme provides forward secrecy and backward secrecy.

## 8. Performance Analysis of Proposed Scheme

In this section, we compare the performance of the proposed scheme with recent studies [12,13,14,15,16,17,18,19], focusing on minimizing the computational overhead of IoT devices while evaluating our scheme against existing works.

For comparison, we utilized computational overhead measurements obtained from a prior study conducted in a CPU-based environment using an Intel Core i7-8700 (3.20 GHz) processor, Windows 10 (64-bit) OS, and 48 GB of memory, employing the Python cryptography library 3.13.2 [33]. The results are presented in Table 10.

In the study by Wang et al. [12], the user performs 8 hash function computations, 1 fuzzy extractor operation, and 3 elliptic curve multiplications, which can be expressed as 8h+1f+3m. Based on the experimental environment, this corresponds to 2129.52 μs. At the cloud center, 10 hash function computations and 1 elliptic curve multiplication are performed, expressed as 10h+1m=533.9 μs. The gateway node performs 9 hash function computations, represented as 9h=1.71 μs. Additionally, the sensor node executes 4 hash function computations and 2 elliptic curve multiplications, which can be expressed as 4h+2m=1064.76 μs.

Wazid et al. [13] introduced an approach where the user carries out 13h+2s+1f, which corresponds to 535.01 μs. Their scheme does not involve a cloud center, so the gateway node handles 5h+4s, taking 2.03 μs. On the sensor side, 4h+2s is performed, resulting in 1.3 μs.

Yang et al. [14]’s method focuses on lightweight computation, requiring the user to compute 8h, leading to 1.52 μs. The gateway node processes 14h, with an execution time of 2.66 μs, while the sensor node performs 7h, taking 1.33 μs.

Srinivas et al. [15] proposed an approach where the user performs 2c+15h+1f, consuming 542.45 μs. Their method involves computations at the gateway node, where 10h is executed, taking 1.9 μs. The sensor node carries out 2c+6h, leading to a processing time of 8.74 μs.

Wazid et al.’s scheme in [16] follows a slightly different structure. The user executes 9h+1s+1f, with a computation time of 533.98 μs. The gateway node computes 11h+2s, requiring 2.63 μs, while the sensor node carries out 7h+1s, taking 1.6 μs.

Dai et al. [17] introduced a model where the user must perform 1f+9h+3m, consuming 2129.71 μs. The gateway node handles 11h+1m, with an execution time of 534.09 μs, and the sensor node processes 5h+2m, leading to 1064.95 μs.

Hu et al. [18]’s scheme assigns 15h+1f+2c computations to the user, requiring 542.45 μs. Their method does not include cloud center processing, so the gateway node executes 7h+1s, with a computation time of 1.6 μs. Meanwhile, the sensor node performs 4h+2c+1s, resulting in 8.63 μs.

Jiang et al. [19] designed a method where the user processes 1f+11h+5m+1s, which takes 3194.36 μs. The cloud center, in their model, executes 5m+1s+12h, consuming 2662.55 μs, while the sensor node handles 4h, leading to 0.76 μs.

In our proposed scheme, as demonstrated in Table 11, we optimize the computational cost across all entities. The user performs 9h+1f+4m, leading to 2661.71 μs. Our approach eliminates unnecessary computations at the cloud center, processing 2m+17h, taking 1067.23 μs. The gateway node handles 9h, requiring 1.71 μs, while the sensor node executes 3h, resulting in a final processing time of 0.57 μs. Our proposed scheme increases the computational load at the cloud center and user mobile devices, thereby reducing the computational burden on the sensor node. While the average computational cost at the sensor node in other schemes is 202.5563 μs, our scheme reduces this to 0.57 μs, making it approximately 35,436.18% more efficient. The overall efficiency of the authentication scheme is most affected by the load and cost of the sensor nodes. Relatively speaking, cloud centers and user mobile devices have relatively high computing resources compared to sensor nodes and gateways, so the increase in computation does not have a significant impact on the overall efficiency of the authentication scheme. Moreover, as demonstrated in Table 9, our proposed scheme ensures stronger security performance compared to other studies.

## 9. Conclusions

In this study, we analyzed Wang et al.’s authentication scheme and identified several limitations, including the vulnerability in illegal session key exchanges due to stored information in IoT devices and publicly transmitted data, as well as inefficiencies despite utilizing cloud centers. To overcome these issues, we incorporated PUF technology into IoT devices to prevent unauthorized session key exchanges and improve efficiency by ensuring that public-key computations are performed exclusively in the cloud center. In addition to safeguarding patient privacy and maintaining untraceability, IoT devices utilize only a temporary $T.SID_{j}$ during authentication and key distribution. In this study, computationally intensive operations are offloaded to the cloud to significantly reduce the computational and energy burden on sensor nodes. This approach enables stable service provision even in resource-constrained medical IoT devices. By performing all authentication and key exchange processes through the cloud center, the proposed scheme achieves lightweight operation at both sensor nodes and gateways. The system is designed under the assumption that the cloud center possesses sufficient computational power and communication resources. In practical deployments, ensuring appropriate cloud resource provisioning and adopting efficient resource management strategies will help maintain system efficiency and stability. Through this approach, the proposed authentication and key distribution scheme is expected to maintain efficiency and robustness across diverse environments. Although the proposed authentication and key distribution scheme is designed for medical IoT environments, its structure is not application-specific, allowing for flexible adaptation to other IoT domains. For instance, the lightweight sensor node design and cloud-based authentication framework can be readily applied to smart homes, industrial IoT, and smart city environments. Therefore, the proposed scheme can maintain high efficiency and stability not only in healthcare but also in a variety of large-scale IoT deployments. Finally, we compared the security and efficiency of our scheme with recent authentication schemes [12,13,14,15,16,17,18,19] and validated its security using ProVerif, a formal security analysis tool.

## Figures and Tables

**Table 1:** Notation.

Notation	Description
$U_{i},$ $SN_{j}$, $GWN_{k}$	*i*-th user, *j*-th IoT device (sensor), *k*-th medical gateway
$UID_{i}$, $PW_{i}$	*i*-th user’s identity and password
$SID_{j}$, $GID_{k}$	*j*-th IoT device’s identity, *k*-th medical gateway’s identity
$T.SID_{j}$	Temporary identity of *j*-th IoT device (sensor)
SC	Long-term secret information of cloud center
$SC_{k}$	Long-term secret information of *k*-th medical gateway
*P*, *d*, *G*	ECC-based public key, private key, base point of cloud center
$(Cn_{c},$ $Rn_{c})$	Challenge–response pair of PUF for cloud center
$(Cn_{g},$ $Rn_{g})$	Challenge–response pair of PUF for gateway
SK	Session key
$R_{1},$ $R_{2},$ …	Random number
$T_{1},$ $T_{2},$ …	Time stamp

**Table 2:** Definitions of channels, variables, and other related parameters.

(*—-channels—-* ^1^)
free privateChannel: channel [private].
free publicChannel: channel.
(*—-constants—-*)
free UserSigma: bitstring [private].
free UserPassword: bitstring [private].
free UserID: bitstring [private].
free GatewayKey: bitstring [private].
free GatewayID: bitstring.
free ServerID: bitstring.
(*—-shared key—-*)
free SharedKey: bitstring [private].
(*—-functions—-*)
fun xor(bitstring, bitstring): bitstring.
fun concat(bitstring, bitstring): bitstring.
fun h(bitstring): bitstring.
fun scalar_mult(bitstring, bitstring): bitstring.
(*—-events—-*)
event startUser(bitstring).
event endUser(bitstring).
event startCloudCenter().
event endCloudCenter().
event startGateway(bitstring).
event endGateway(bitstring).
event startSensorNode(bitstring).
event endSensorNode(bitstring).

^1^ In ProVerif, the (*…*) notation indicates a comment.

**Table 3:** User process.

(*—-User Process—-*)
let UserProcess = let checkUserID = xor(h(concat(UserID, UserPassword)), h(UserSigma)) in
out(privateChannel, (UserID, UserSigma));
in(privateChannel, (receivedUserID: bitstring, receivedA: bitstring, receivedP: bitstring, receivedG: bitstring));
if receivedUserID = UserID then
let computedR1 = xor(receivedA, h(concat(UserID, UserSigma))) in
new R2: bitstring;
let computedB = xor(h(concat(UserID, UserSigma)), R2) in
let computedC = h(concat(UserID, R2)) in
event startUser(UserID);
new inputUserID:bitstring;
new inputUserPassword:bitstring;
new inputUserSigma:bitstring;
if checkUserID = xor(h(concat(inputUserID, inputUserPassword)), h(inputUserSigma)) then
let computedR2 = xor(computedB, h(xor(inputUserID,inputUserSigma))) in
if computedC = h(concat(inputUserSigma, computedR2)) then
new R3:bitstring;
new T1:bitstring;
new SensorID:bitstring;
let computedM1 = scalar_mult(R3, receivedP) in
let computedM2 = scalar_mult(R3, receivedG) in
let computedM3 = xor(xor(h(concat(computedM1,computedM2)), computedR1), h(concat(inputUserID,inputUserSigma))) in
let computedM4 =xor( h(xor(xor(computedM1,computedM2), computedM3)), SensorID) in
let computedM5 =h(concat(concat(concat(computedR1,computedM1),computedM2),SensorID)) in
out(publicChannel, (inputUserID,computedM2, computedM3, computedM4, computedM5, T1 ));
in(publicChannel, (receivedM14: bitstring, receivedM15: bitstring, receivedM16: bitstring, receivedT6: bitstring));
let SharedKey = xor(xor(receivedM14,h(concat(concat(computedM1,computedR1),receivedT6))),
h(concat(inputUserID,inputUserSigma))) in
if scalar_mult(SharedKey,receivedP) = scalar_mult(computedM5,receivedG) then
let computedM16 = h(concat(concat(concat(SharedKey,computedM1),receivedM15),receivedT6)) in
if computedM16 = receivedM16 then
event endUser(UserID).

**Table 4:** CloudCenter process.

(*—-CloudCenter Process—-*)
let CloudCenterProcess = in(privateChannel, (receivedUserID:bitstring, receivedSigma:bitstring));
new R1:bitstring;
new P:bitstring;
new G:bitstring;
let computedA = xor(h(concat(receivedUserID,receivedSigma )),R1) in
out(privateChannel, (receivedUserID, computedA, P, G));
in(privateChannel, (receivedGatewayID:bitstring));
new SC:bitstring;
let computedXG=h(concat(SC,receivedGatewayID )) in
out(privateChannel, (receivedGatewayID, computedXG));
in(privateChannel, (receivedCheckG:bitstring));
in(privateChannel, (receivedSensorID:bitstring));
new Challenge:bitstring;
new TempSensorID:bitstring;
out(privateChannel, (Challenge,TempSensorID ));
in(privateChannel,(receivedRNC:bitstring));
event startCloudCenter();
in(publicChannel, (receivedUserID:bitstring, receivedM2:bitstring, receivedM3:bitstring,
receivedM4:bitstring, receivedM5:bitstring, receivedT1:bitstring));
new d:bitstring;
let computedM1=scalar_mult(d, receivedM2) in
let computedR1=xor(xor(receivedM3,h(concat(computedM1,receivedM2))),h(concat(receivedUserID,receivedSigma ))) in
if computedR1= xor(computedA, h(concat(receivedUserID, receivedSigma))) then
let computedSensorID = xor(receivedM4, h(concat(concat(computedM1,receivedM2 ),receivedM3))) in
if computedSensorID=receivedSensorID then
if receivedM5=h(concat(concat(concat(xor(h(concat(receivedUserID,receivedSigma)),
computedR1),computedM1),receivedM2),receivedSensorID)) then
new SK:bitstring;
new T2:bitstring;
let computedM6 = xor(xor(SK,h(computedXG)),h(concat(receivedCheckG,computedXG ))) in
let computedM7 = xor(h(concat(concat(SK,computedM6),computedXG)),TempSensorID) in
let computedM8 =h(concat(concat(concat(concat(SK,computedM6),computedXG),TempSensorID),T2)) in
out(publicChannel,(TempSensorID,computedM6, computedM7, computedM8, Challenge, T2 ));
in(publicChannel, (receivedTempSensorID:bitstring, receivedM12:bitstring, receivedM13:bitstring, receivedT5:bitstring));
let computedM12= h(concat(SK,h(receivedRNC))) in
let computedM13= xor(h(concat(SK,computedM12)),h(concat(receivedT5,computedXG))) in
if computedM12=receivedM12 then
if computedM13=receivedM13 then
new T6:bitstring;
let computedM14 = xor(xor(SK,h(concat(concat(computedM1,computedR1),T6))),h(concat(receivedUserID,receivedSigma))) in
let computedM15 =scalar_mult(SK, d) in
let computedM16 = h(concat(concat(concat(SK,computedM1),computedM15),T6)) in
out(publicChannel, (computedM14, computedM15,computedM16, T6 ));
event endCloudCenter().

**Table 5:** Gateway process.

(*—-Gateway Process—-*) let GatewayProcess = new GatewayID:bitstring;
out(publicChannel, (GatewayID));
in(publicChannel,(receivedGatewayID:bitstring, receivedXG:bitstring));
new SG:bitstring;
let computedCheckG =h(concat(SG,receivedXG)) in
out(publicChannel, (computedCheckG));
in(publicChannel, (receivedSensorID:bitstring));
new CNg:bitstring;
out(publicChannel, (CNg));
in(publicChannel, (receivedSensorID:bitstring, receivedTempSensorID:bitstring, receivedRN:bitstring));
event startGateway(GatewayID);
in(publicChannel, (receivedTemporSensorID:bitstring, receivedM6:bitstring, receivedM7:bitstring,
receivedM8:bitstring, receivedCNc:bitstring, receivedT2:bitstring));
let computedSK = xor(xor(receivedM6,h(receivedXG)),h(concat(computedCheckG,receivedXG))) in
let TempSensorID = xor(receivedM7, h(concat(concat(computedSK,receivedM6),receivedXG))) in
if receivedM8 = h(concat(concat(concat(concat(computedSK,receivedM6),receivedXG),receivedTemporSensorID),receivedT2)) then
new T3:bitstring;
let computedRNg= xor(xor(SG,receivedRN),SG) in
let computedM9 = xor(computedSK, h(receivedRN)) in
let computedM10 = h(concat(concat(concat(computedSK,receivedTemporSensorID),receivedRN),T3))in
out(publicChannel, (GatewayID,computedM9, computedM10, receivedCNc, CNg, T3 ));
in(publicChannel, (receivedTempSensorID:bitstring, receivedM11:bitstring, receivedM12:bitstring, receivedT4:bitstring));
if receivedM11 =h(concat(concat(computedSK,h(receivedRN)),receivedT4)) then
new T5:bitstring;
let computedM13=xor(h(concat(computedSK,receivedM12)), h(concat(T5,receivedXG))) in
out(publicChannel, (receivedTempSensorID,receivedM12, computedM13, T5));
event endGateway(GatewayID).

**Table 6:** SensorNode process.

(*—-SensorNode Process—-*)
let SensorNodeProcess = new SensorID:bitstring;
out(publicChannel, (SensorID));
in(publicChannel, (receivedCn:bitstring, receivedTempSensorID:bitstring));
new RNc:bitstring;
out(publicChannel, (RNc));
out(publicChannel, (SensorID));
in(publicChannel, (CNg:bitstring));
new RNg:bitstring;
out(publicChannel, (SensorID,receivedTempSensorID, RNg ));
event startSensorNode(SensorID);
in(publicChannel, (receivedGatewayID:bitstring, receivedM9:bitstring, receivedM10:bitstring,
receivedCNc:bitstring, receivedCNg:bitstring, receivedT3:bitstring));
new RNg:bitstring;
let computedSK = xor(receivedM9,h(RNg)) in
if receivedM10 = h(concat(concat(concat(computedSK,receivedTempSensorID),RNg),receivedT3)) then
new T4:bitstring;
new RNc:bitstring;
let computedM11 = h(concat(concat(computedSK,h(RNg)),T4)) in
let computedM12 = h(concat(computedSK,h(RNc)))in
out(publicChannel, (receivedTempSensorID, computedM11,computedM12, T4 ));
event endSensorNode(SensorID).

**Table 7:** Queries and main process.

(*—-queries—-*)
query idi:bitstring; inj-event(endUser(idi)) ==> inj-event(startUser(idi)).
query inj-event(endCloudCenter()) ==> inj-event(startCloudCenter()).
query gwi:bitstring; inj-event(endGateway(gwi)) ==> inj-event(startGateway(gwi)).
query snj:bitstring; inj-event(endSensorNode(snj)) ==> inj-event(startSensorNode(snj)).
query attacker(SharedKey).
(*—-process—-*)
process ((!UserProcess)|(!CloudCenterProcess)|(!GatewayProcess)|(!SensorNodeProcess))

**Table 8:** Result.

Verification summary:
Query inj-event(endUser(idi)) ==> inj-event(startUser(idi)) is true.
Query inj-event(endCloudCenter) ==> inj-event(startCloudCenter) is true.
Query inj-event(endGateway(gwi)) ==> inj-event(startGateway(gwi)) is true.
Query inj-event(endSensorNode(snj)) ==> inj-event(startSensorNode(snj)) is true.
Query not attacker(SharedKey[]) is true.

**Table 9:** Comparison of security features.

Security Features	Wang et al. [12]	Wazid et al. [13]	Yang et al. [14]	Srinivas et al. [15]	Wazid et al. [16]	Dai et al. [17]	Hu et al. [18]	Jiang et al. [19]	Ours
A1	O	O	O	O	O	O	O	O	O
A2	O	O	O	O	O	O	O	O	O
A3	X	O	O	O	O	O	O	O	O
A4	O	O	O	O	O	O	O	O	O
A5	O	O	O	O	O	O	O	O	O
A5	O	O	O	O	O	O	O	O	O
S1	O	O	O	X	O	O	O	O	O
S2	O	O	O	O	O	O	O	O	O
S3	O	X	X	O	X	O	O	O	O

**Table 10:** Computation times for each operation (μs).

Symbol	Meaning	Time (μs)
*h*	Computation time for hash functions	0.19
*f*	Extraction time of biometric information in fuzzy extractors	532
*m*	Multiplication operation time in ECC	532
*s*	Computation time for symmetric key encryption or decryption	0.27
*c*	Chebyshev polynomial computation time	3.8

**Table 11:** Comparisons of computational costs (μs).

Scheme	User	Cloud Center	Gateway Node	Sensor Node
Wang et al. [12]	8h+1f+3m	10h+1m	9h	4h+2m
Wazid et al. [13]	13h+2s+1f	-	5h+4s	4h+2s
Yang et al. [14]	8h	-	14h	7h
Srinivas et al. [15]	2c+15h+1f	-	10h	2c+6h
Wazid et al. [16]	9h+1s+1f	-	11h+2s	7h+1s
Dai et al. [17]	1f+9h+3m	-	11h+1m	5h+2m
Hu et al. [18]	15h+1f+2c	-	7h+1s	4h+2c+1s
Jiang et al. [19]	1f+11h+5m+1s	5m+1s+12h	-	4h
Ours	9h+1f+4m	2m+17h	9h	3h

## Data Availability

Data are contained within the article.
